# [Retracted] ABCG1 as a potential oncogene in lung cancer

**DOI:** 10.3892/etm.2025.12858

**Published:** 2025-04-01

**Authors:** Chunyan Tian, Di Huang, Yanli Yu, Jihong Zhang, Qingxin Fang, Chao Xie

Exp Ther Med 13:3189–3194, 2017; DOI: 10.3892/etm.2017.4393

Following the publication of this paper, it was drawn to the Editor’s attention by a concerned reader that certain of the western blot data shown in Fig. 1A on p. 3191 and the cell migration assay data in Fig. 5 on p. 3193 were strikingly similar to data appearing in different form in other articles written by different authors at different research institutes that had already been published elsewhere prior to the submission of this paper to *Experimental and Therapeutic Medicine*. In view of the fact that the abovementioned data had already apparently been published previously, the Editor of *Experimental and Therapeutic Medicine* has decided that this paper should be retracted from the Journal. The authors were asked for an explanation to account for these concerns, but the Editorial Office did not receive a reply. The Editor apologizes to the readership for any inconvenience caused.

